# Appearances of the Tongue in Disease

**Published:** 1831

**Authors:** 


### APPEARANCES OF THE TONGUE IN DISEASE. By M. PIORRY.

IN all cases in which the pulse is strong, frequent, full and developed, the colour of the tongue, in common with that of the conjunctiva, cheeks, lips, pharynx, and gums, is red. After extensive evacuations of blood, and in chronic affections, all the tissues are pale, and consequently the tongue also. In cases of gastritis, enteritis, and dysentery, attended with trifling fever, the colour of the tongue is more or less pale. In traumatic fever, and in pneumonia, uncomplicated with gastric symptoms, the tongue is generally of a vermilion, sometimes of a deep red; after repeated bleedings, however, or when the stomach and liver become consecutively diseased, the tongue becomes pale. Most generally, the increased redness of the tongue is perceptible only at its edges, the middle being variously coloured, in consequence of the coating which it assumes, but when the latter is removed, the colour of the whole organ is found to be uniform. The apex of the tongue is not often reddened, excepting during the efforts which the patient makes to protrude the organ; as soon as these efforts cease the redness of this part disappears. The dryness of the tongue's surface is produced by the evaporation of the fluids by which it is covered. Every affection, therefore, which causes respiration to be effected by the mouth, tends to produce this dryness. It occurs in coryza, in all diseases affecting the nasal fossæ, and in cases attended with accelerated respiration; hence the tongue is peculiarly dry in intense pneumonia, especially when attended with coryza, and in pleurisy. Fever, accompanied with increased frequency in the contractions of the heart, and consequently with frequent respiration; diseases of the liver, stomach and peritoneum, in which the respiration is accelerated by the full descent of the diaphragm being prevented, are attended with a dryness of the tongue.

The repeated observations and experiments of M. PIORRY, in relation to the saliva and mucus of the mouth, have convinced him that the various coatings of the tongue and teeth met with in disease are produced by different degrees of exsiccation in the fluids, by which these parts are uniformly covered.

---

